# Hospital Coordination and Protocols Using Serum and Peripheral Blood Cells from Patients and Healthy Donors in a Longitudinal Study of Guillain–Barré Syndrome

**DOI:** 10.3390/diagnostics15151900

**Published:** 2025-07-29

**Authors:** Raquel Díaz, Javier Blanco-García, Javier Rodríguez-Gómez, Eduardo Vargas-Baquero, Carmen Fernández-Alarcón, José Rafael Terán-Tinedo, Lorenzo Romero-Ramírez, Jörg Mey, José de la Fuente, Margarita Villar, Angela Beneitez, María del Carmen Muñoz-Turrillas, María Zurdo-López, Miriam Sagredo del Río, María del Carmen Lorenzo-Lozano, Carlos Marsal-Alonso, Maria Isabel Morales-Casado, Javier Parra-Serrano, Ernesto Doncel-Pérez

**Affiliations:** 1 TOSGB Biobank Collection, Biobanco del Hospital Universitario de Toledo (BioB-HUT), Servicio de Salud de Castilla-La Mancha (SESCAM), Avenida del Río Guadiana, 45007 Toledo, Spain; rdiazmartinez@yahoo.com; 2 Hospital Nacional de Parapléjicos, Servicio de Salud de Castilla-La Mancha (SESCAM), 45071 Toledo, Spain; jablga@yahoo.es (J.B.-G.); jarogo@sescam.jccm.es (J.R.-G.); evargas@sescam.jccm.es (E.V.-B.); mfdezalarcon@gmail.com (C.F.-A.); jteran@sescam.jccm.es (J.R.T.-T.); lromeroramirez@sescam.jccm.es (L.R.-R.); jmey@sescam.jccm.es (J.M.); 3 Instituto de Investigación Sanitaria de Castilla-La Mancha (IDISCAM), 45004 Toledo, Spain; 4 SaBio, Instituto de Investigación en Recursos Cinegéticos (IREC), Consejo Superior de Investigaciones Científicas (CSIC), Universidad de Castilla-La Mancha (UCLM)-Junta de Comunidades de Castilla-La Mancha (JCCM), Ronda de Toledo 12, 13005 Ciudad Real, Spain; jose_delafuente@yahoo.com (J.d.l.F.); margaritam.villar@uclm.es (M.V.); 5 Department of Veterinary Pathobiology, Center for Veterinary Health Sciences, Oklahoma State University, Stillwater, OK 74078, USA; 6 Biochemistry Section, Faculty of Science and Chemical Technologies, University of Castilla-La Mancha, 13071 Ciudad Real, Spain; 7 Centro Regional de Transfusiones Toledo-Guadalajara, Servicio de Salud de Castilla-La Mancha (SESCAM) Avenida de Barber, 30, 45004 Toledo, Spain; abeneitezf@sescam.jccm.es (A.B.); mmturrillas@sescam.jccm.es (M.d.C.M.-T.); 8 Laboratorio de Bioquímica Clínica, Hospital General Universitario de Toledo, Servicio de Salud de Castilla-La Mancha (SESCAM), Avenida del Río Guadiana, 45007 Toledo, Spain; mariazurdo96@gmail.com (M.Z.-L.); msagredo@sescam.jccm.es (M.S.d.R.); mcarmenll@sescam.jccm.es (M.d.C.L.-L.); 9 Servicio de Neurología, Hospital General Universitario de Toledo, Servicio de Salud de Castilla-La Mancha (SESCAM), Avenida del Río Guadiana, 45007 Toledo, Spain; cmarsal05@gmail.com (C.M.-A.); mabelmc3006@gmail.com (M.I.M.-C.); javi.parraserrano@gmail.com (J.P.-S.)

**Keywords:** biomarkers, biobank collection, diagnosis, Guillain–Barre syndrome, transcriptomics, proteomics, biochemical parameters, phenotyping

## Abstract

**Background/Objectives:** Guillain–Barré syndrome (GBS) is a rare autoimmune peripheral neuropathy that affects both the myelin sheaths and axons of the peripheral nervous system. It is the leading cause of acute neuromuscular paralysis worldwide, with an annual incidence of less than two cases per 100,000 people. Although most patients recover, a small proportion do not regain mobility and even remain dependent on mechanical ventilation. In this study, we refer to the analysis of samples collected from GBS patients at different defined time points during hospital recovery and performed by a medical or research group. **Methods:** The conditions for whole blood collection, peripheral blood mononuclear cell isolation, and serum collection from GBS patients and volunteer donors are explained. Aliquots of these human samples have been used for red blood cell phenotyping, transcriptomic and proteomic analyses, and serum biochemical parameter studies. **Results:** The initial sporadic preservation of human samples from GBS patients and control volunteers enabled the creation of a biobank collection for current and future studies related to the diagnosis and treatment of GBS. **Conclusions:** In this article, we describe the laboratory procedures and the integration of a GBS biobank collection, local medical services, and academic institutions collaborating in its respective field. The report establishes the intra-disciplinary and inter-institutional network to conduct long-term longitudinal studies on GBS.

## 1. Introduction

Guillain–Barré syndrome (GBS) is a rare, acute autoimmune disorder affecting the peripheral nervous system (PNS), and is currently recognized as the leading cause of acute neuromuscular paralysis worldwide, with an annual incidence of 1.12 cases per 100,000 individuals [1]. GBS often arises following bacterial infections such as Campylobacter jejuni, viral infections including HIV-1; hepatitis C, Zika and Chikungunya viruses; or as a post-infectious complication associated with COVID-19 [2,3,4,5,6]. Despite its clinical importance, early diagnosis of GBS remains challenging due to its variable presentation and symptom overlap with other neurological disorders.

Accurate diagnosis typically involves a comprehensive medical history and physical examination, supported by diagnostic tests such as lumbar puncture, used to detect elevated protein levels in cerebrospinal fluid [7]; electromyography (EMG) to assess muscle response; and nerve conduction studies to evaluate the speed and integrity of peripheral nerve signaling [8].

Currently, there is no definitive cure for GBS. Treatment strategies focus on immunomodulatory interventions such as plasmapheresis, which removes circulating antibodies contributing to the autoimmune attack, and intravenous immunoglobulin (IVIG) therapy, which provides passive immunization to neutralize pathogenic antibodies [9,10]. However, both treatments show variable efficacy, and a subset of patients may experience severe progression requiring intensive care and long-term rehabilitation [10]. The average cost of hospital treatment per patient is about €950 for plasmapheresis compared to €1889 for IVIG [11], and increases significantly with ICU admission and longer recovery periods.

In this study, the diagnosis of GBS was based on case-specific criteria aligned with the updated guidelines of the National Institute of Neurological Disorders and Stroke (NINDS). These diagnostic criteria were systematically applied by trained neurologists at a general hospital when GBS was clinically suspected. Key criteria included (1) progressive weakness in the limbs—ranging from mild lower-limb weakness to complete quadriplegia—including involvement of truncal, bulbar, and facial muscles, as well as external ophthalmoplegia; (2) areflexia or diminished deep tendon reflexes in affected limbs; (3) progression of symptoms over a period of ≤4 weeks; (4) electrodiagnostic findings consistent with GBS, such as prolonged or absent F waves, absent H reflexes, prolonged distal latencies, conduction blocks with temporal dispersion, significantly slowed nerve conduction velocities, and reduced recruitment or denervation on needle electromyography in weak muscles; and (5) cerebrospinal fluid (CSF) abnormalities, including albuminocytologic dissociation—characterized by elevated protein levels (45–200 mg/dL [0.45–2.0 g/L]) with a normal white blood cell count (<5 cells/mm^3^, though occasionally up to 50 cells/mm^3^). Additional supportive criteria as outlined by the NINDS were also considered where applicable [12,13,14,15,16].

Following informed consent, biospecimens such as serum and peripheral blood mononuclear cells (PBMCs) are obtained from these patients and stored in the hospital’s TOSGB Biobank Collection (in Spanish, TOSGB: Toledo, Síndrome de Guillaín Barré). The present article describes the collaborative framework to evaluate GBS patients in Castilla-La Mancha, Spain (BioGBS project) and first results to validate previously described GBS biomarkers [17,18]. We have performed a retrospective and longitudinal analysis of biological samples from patients with GBS and matched controls. This study highlights the central role of the biobank in integrating transcriptomic, proteomic, biochemical, and immunophenotypic data to advance our understanding of GBS pathophysiology and identify potential biomarkers related to disease severity, progression, and therapeutic targets.

## 2. Materials and Methods

### 2.1. GBS Patients and Controls

The use of human material, including PBMC and peripheral blood serum samples from GBS, traumatic spinal cord injury (tSCI) patients, and healthy individuals, was approved by the Clinical Research Ethics Committee for Hospitals of Toledo City, Castilla-La Mancha, Spain (permit number 17 in support information), and informed consent was obtained from all individuals in compliance with the Helsinki Declaration. Blood samples of patients and controls were extracted by nursing personnel in the Hospital Nacional de Parapléjicos; and Centro Regional de Transfusiones, bloodbank (Toledo, Spain).

### 2.2. Whole Blood Processing for PBMCs and Serum

The general protocol for human peripheral blood mononuclear cells (PBMCs) isolation has been reported elsewhere [19]. In our PBMCS isolation conditions, EDTA-anticoagulated whole blood was diluted by half in cold PBS. The diluted blood was carefully decanted, avoiding mixing with Ficoll (Cytiva, Uppsala, Sweden) at a ratio of 10:3, volume/volume. After Ficoll gradient centrifugation at 660 g for 30 min at room temperature, a ring of peripheral blood mononuclear cells (PBMCs) was obtained. The PBMCs pellet was resuspended in nucleic acid preservative solution (TRIzol; Invitrogen, Madrid, Spain), aliquoted, coded, and stored at −80° in TOSGB Biobank collection until used for RNA extraction.

For serum isolation, approximately 8 mL of whole blood was collected in a sterile gel-barrier tube (FL Medical, Padua, Italy). This kind of tube was used to separate serum from the blood clot, as the gel barrier ascends toward the serum clot during centrifugation, for 10 min at 2650× *g*. The serum was collected, coded, and stored at −80° in TOSGB Biobank collection until used for various analyses.

### 2.3. Transcriptomics and Real Time RT-PCR of PBMCs

These two procedures were previously described by us [12]. Briefly, RNA was extracted from PBMCs in TRIzol and its quality was assessed, showing high RNA integrity. Libraries were prepared using the TruSeq RNA Kit, and the expected fragment size was confirmed using a bioanalyzer. Sequencing was performed on an Illumina GAIIx platform, with a throughput of between 13.4 and 15.7 million 75-base single-end reads per sample. Sequencing data were deposited in Gene Expression Omnibus (accession no. GSE72748). Transcript abundance was quantified using Cufflinks, and differentially expressed genes were identified with Cuffdiff, applying strict significance thresholds. Gene ontology analysis was performed using Blast2GO to interpret functional implications.

Further validation of selected genes, including *EGR1*, *EGR2*, and *GBP1*, was performed through real-time RT-PCR using TaqMan probes and reference genes with stable expression profiles. The expression of *Clorf31* was also analyzed using SYBR Green chemistry and primers designed via Primer-BLAST. Normalization was conducted using reference genes, and relative expression levels were determined through the ΔΔCt method. Statistical analysis involved t-tests with appropriate corrections for multiple comparisons to ensure robustness of the gene expression data.

### 2.4. Proteomic Analysis of Human Serum

Our group previously described data acquisition and proteomic analysis in detail [13]. Briefly, serum samples from GBS and tSCI patients were analyzed to identify differential protein production. Proteins were extracted, separated by SDS-PAGE, and the concentrated proteins were processed by Coomassie staining, reduction, alkylation, and enzymatic digestion with trypsin. The resulting peptides were labeled using the iTRAQ 8plex kit (Applied Biosystems, Foster City, CA, USA), pooled, desalted, and analyzed via reverse-phase liquid chromatography coupled with high-resolution tandem mass spectrometry (RP LC-MS/MS) on an Orbitrap system. A top-20 data-dependent acquisition method with HCD fragmentation was used to generate MS/MS spectra.

Data were processed with Proteome Discoverer and searched against the Uniprot human database; Thermo Proteome Discoverer 1.4, with a Uniprot database containing 147.854 entries of *Homo sapiens* (8 September 2015), and using specific modifications of iTRAQ. Peptides were also matched against reversed databases to control for false discovery rates (FDR ≤ 5%). Quantitative data were obtained using in-house developed program, QuiXoT software [20], and changes in protein levels were assessed using the Generic Integration Algorithm [21]. Statistical analysis was performed at spectrum, peptide, and protein levels using the WSPP model [20] to determine significance and ensure data reliability.

### 2.5. Biochemical Analysis in Human Serum

Serum samples were received at the biochemistry laboratory on ice and kept refrigerated while thawing, until processing, which in most cases was carried out on the same day of reception. The biochemical analysis included the determination of 14 parameters using spectrophotometric techniques on the cobas c 702 analyzer (Roche^®^; Barcelona, Spain). Interleukin 6 (IL-6) was quantified by immunoassay using the cobas e 801 analyzer (Roche^®^). Meanwhile, the concentrations of sodium (Na^+^), potassium (K^+^), and chloride (Cl^−^) ions were determined by indirect potentiometry using the ion-selective electrode (ISE) module of the cobas c 702. The specific methodology for each biochemical and ionic parameters present in the analyzed samples are explained in following Table 1.

### 2.6. Phenotyping in Red Blood Cells

Red blood cell phenotyping was conducted to determine ABO and Rh blood groups. Red blood cell phenotyping was performed to determine ABO and Rh blood groups, extended red cell antigen profiles, the presence of irregular antibodies, and the result of the Direct Coombs test. Whole blood samples were collected in EDTA tubes, and analyses were performed using specific antisera with agglutination techniques in microplate, gel column, or microsphere-based platforms, employing various commercial systems (Werfen, Barcelona, and Bio-Rad, Madrid, Spain; and QuidelOrtho, San Diego, CA, USA).

ABO and RhD typing were conducted using an automated microplate method with monoclonal IgM reagents. Samples initially typed as RhD negative were further tested with a second, more specific antiserum to detect possible weak or partial RhD variants. Extended phenotyping included additional Rh antigens (C, c, E, e), as well as antigens from other blood group systems, such as Kell, Duffy, Kidd, MNS, Lutheran, Lewis, and P1. Reagents used for each antigen were either IgG or IgM, depending on the manufacturer’s recommendations. For irregular antibody screening, patient plasma was incubated with commercial screening red cells using Immucor’s Capture microplate technology (Norcross, GA, USA) to identify unexpected alloantibodies. The Direct Coombs test was performed by adding anti-human globulin reagent to the patient’s red blood cells to detect in vivo coating with immunoglobulin or complement. Agglutination patterns in gel matrices or microplates were interpreted automatically by the analyzers and visually confirmed by laboratory personnel, ensuring accurate and comprehensive immunohematologic profiling.

### 2.7. Statistical Analysis

Each experimental assay was performed with replicates. The reproducibility of biochemical or phenotyping results is based on consistent diagnostic values guaranteed by the local protocol or the equipment of the aforementioned brand.

## 3. Results

### 3.1. Recruitment of Participants in BioGBS Study and Identification of GBS Subtypes

**Figure 1:**
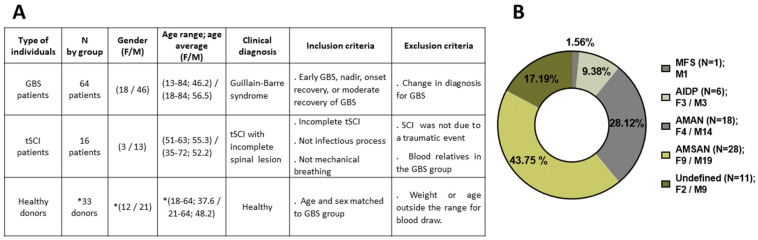
**Patient and control individuals included in the current BioGBS study**. In (**A**): composition of each group with demographic, clinical diagnosis, and inclusion/exclusion criteria for the BioGBS study; (*) number of healthy donors increases progressively until reaching the same number of GBS group for stratification by age and sex. In (**B**): current distribution of GBS Subtypes in GBS Biobank Collection: MFS, Miller Fisher Syndrome; AIDP, Acute Inflammatory Demyelinating Polyradiculoneuropathy; AMAN, Acute Motor Axonal Neuropathy; AMSAN, Acute Motor Sensory Axonal Neuropathy; and Undefined GBS subtype. N, number of individuals; (F/M) female/male; GBS, Guillain–Barre syndrome; tSCI, traumatic spinal cord injury.

This observational and longitudinal study included 113 individuals, 80 patients and 33 controls. All donors were previously informed about the BioGBS study, and informed consent was signed by the donor or a witness if the donor was unable to sign. Subtypes of Guillain–Barré syndrome (GBS) were obtained in arrival at Hospital Nacional de Parapléjicos (HNP) de Toledo. At the time of writing, the sample size continues to increase to improve stratification by gender and age to match the healthy donor and GBS patient groups (Figure 1).

Pediatric GBS cases were rare but present, whereas GBS appears around middle age and 10 years earlier in women than in men in this local BioGBS study. The traumatic spinal cord injury (tSCI) group had the lowest number of cases and, as a control group, contributes to the normalization of hospitalization conditions, medical care, and rehabilitation program, compared with the GBS group. The number of hospitalized women was lower than that of men in all groups. This could be a sampling artefact due to the low number of GBS patients hospitalized during the study period, a common occurrence in rare diseases (Figure 1A).

In the current distribution of GBS subtypes in GBS Biobank Collection, the AMSAN subtype is the most represented, at 43.75%, followed by AMAN (28.12%), AIDP (9.38%), and MFS (1.56%). Patients with GBS who were not classified by electromyography at the time of admission to the HNP are identified as an undefined group, approximately 17.19%. The number of men affected by GBS was always greater than woman in all identified GBS subtypes (Figure 1B).

### 3.2. Times Points During GBS Disease and Recovery for Collection of Serum and PBMCs

**Figure 2:**
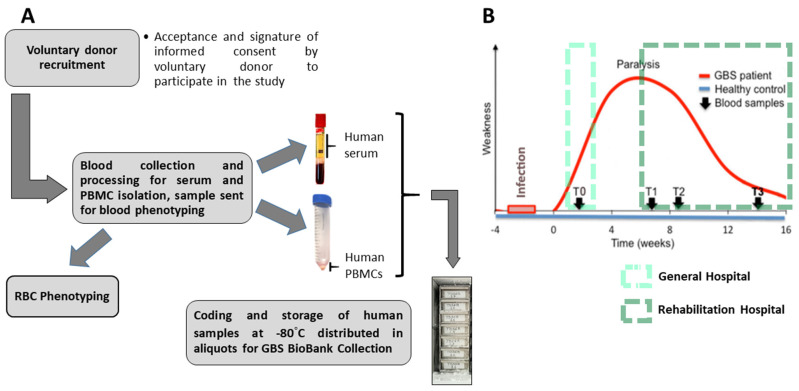
**Whole blood processing from volunteer donors and selected blood collection time points from Guillain–Barre syndrome onset to recovery.** The different steps for obtaining serum, PBMCs, red blood cells (RBC) phenotyping, and final storage of samples in the GBS Biobank Collection (TOSGB) are shown (**A**). Monophasic behavior curve for GBS progression and recovery after infection; blood collections performed in general and rehabilitation hospitals at time points T0, T1, T2, and T3, corresponding to the early, plateau, onset recovery, and moderate recovery phases of GBS (**B**).

After each volunteer donor accepted and signed the informed consent form to participate in the BioGBS study, blood collection and processing were performed. On the day of collection, human serum and human peripheral blood mononuclear cells (PBMCs) pellets were obtained. On the same day as the first collection, an EDTA-anticoagulated blood fraction was sent to the blood bank for erythrocyte phenotyping of each individual. From 9.0 mL of whole blood, between 3.5 and 4.5 mL of human serum were obtained. An initial volume of 27–30 mL of EDTA-anticoagulated blood yields a cell pellet of between 150 and 200 mg of human PBMCs, under our laboratory conditions and using standard methodologies (Figure 2A).

The present BioGBS study included patients with GBS at early stages (T0), plateau stages (T1), early recovery stages (T2), and moderate recovery stages (T3). T0, T1, T2, and T3 represent the time points of blood sampling from the same patient during the development and recovery of GBS. The recovery process varies from patient to patient, necessitating close monitoring of GBS patients to confirm positive changes in their physical recovery (Figure 2B).

GBS patients arrived to the HNP at the lowest stage of their recovery, nadir; with respiratory failure requiring mechanical ventilation (T1). These patients, transferred from a general hospital, did not recover from symptoms of weakness that persisted for more than four weeks (prolonged plateau phase of paralysis). The start of recovery was established when the GBS patient achieved independent breathing (T2). The final time point for blood collection occurred just before hospital discharge, after the GBS patient completed the medical and rehabilitation program at HNP (T3); see Figure 2B.

The Neurology Department of a local collaborating hospital is recruiting donors with early-stage GBS (T0). There, a trained neurologist diagnosed GBS, and nursing staff performed blood draw. Blood is collected from patients with early-stage GBS after diagnosis and before clinical and pharmacological treatment. GBS patients are subsequently followed for outcome, and additional blood draws (T1, T2, and T3) are performed in cases of treatment failure and a prolonged nadir requiring mechanical ventilation until recovery and hospital discharge. Whole blood is processed by the GBS Biobank collection staff according to the procedure described above (Figure 2).

### 3.3. Quantitative Real-Time PCR in PBMCs, Serum Proteomic Analysis, and Serum Antibodies Searching from Samples in GBS Biobank Collection

**Figure 3:**
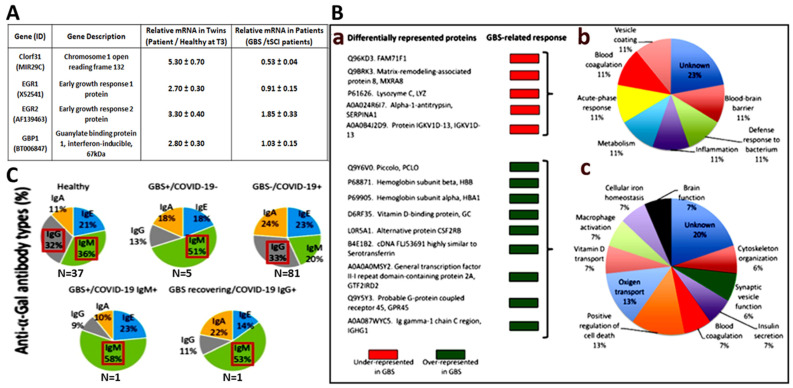
**Examples of quantitative real-time PCR in PBMCs, serum proteomic analysis, and serum antibodies from samples in the TOSGB Biobank collection.** The mRNA from PBMCs was used to determine gene expression levels of GBS-specific genetic markers (**A**). Serum from GBS patients and controls were processed for proteomic analysis (**B**), expression of GBS-specific proteins is shown in the left panel (**a**), and the corresponding affected biological function in the right panel (**b**,**c**). Specific anti-α-Gal antibody types in serum from GBS patients and GBS/COVID-19 patients stored in the GBS Biobank, compared with COVID-19 patients and healthy controls (**C**). Modified from [17,18,22] with permission.

A primary objective of the BioGBS study was to identify markers for GBS progression and recovery [17,18]. To this end, PBMCs were obtained from a GBS patient and her healthy twin; this allowed us to drastically reduce genetic variations since both share the same genetic background. Genetic studies were performed by transcriptomic analysis of PBMCs isolated at the T1-T3 time points of GBS recovery and corroborated by quantitative real-time RT-PCR. The four overexpressed genes obtained in the GBS patient versus her healthy twin were consistent in GBS patients versus tSCI donors used as an internal control [12]. Significantly expressed genes related to GBS recovery are shown (Figure 3A). This gene expression can be validated in PBMC samples from a larger number of GBS patients and controls than in the initial study, which are held in the GBS Biobank collection.

Our group obtained protein markers associated with GBS disease in the serum of GBS patients compared with controls. Nine overexpressed and five underexpressed proteins were identified, a significant protein expression related to GBS [13]. The role of these serum proteins in GBS progression and recovery can be studied in depth using our GBS sample repository (Figure 3B).

Another example of the use of serum samples from the GBS Biobank collection was the search for anti-α-Gal antibody types in GBS, GBS-COVID-19, COVID-19, and healthy individuals [22]. Seven cases diagnosed with GBS and hospitalized during COVID-19 pandemic were included in the study. Of them, two cases were confirmed infected with SARS-CoV-2 by the RT-PCR assay from upper respiratory tract samples after hospital admission. Data from COVID-19 patients (pooled from hospital discharge, N = 27; hospitalized, N = 29; intensive care unit, N = 25), N = 81 total cases, and healthy control individuals (sera collected from individuals with no record of tick bites and allergic reactions before the COVID-19 pandemic in April 2019, N = 37) were obtained from a previously conducted retrospective case-control study [23] (Figure 3C).

Saccharide-induced immune responses were related to GBS and the α-Gal syndrome (AGS). The AGS is a tick-induced allergy to mammalian meat triggered by the IgE antibody response against the carbohydrate Galα1-3Galβ1-(3)4GlcNAc-R (α-Gal), ref. [24]. The study concluded that the decreased IgM/IgG antibody response to α-Gal observed in GBS patients could reflect a dysbiosis of the gut microbiota associated with infection with pathogens that trigger neuropathy, and that GBS should not be considered a factor that increases anti-α-Gal IgE levels and, therefore, the risk of tick-bite-related allergies [22]. This research was made possible through a collaboration between the university research institutions (IREC, UCLM), the Hospital Microbiology Service, and the GBS Biobank collection (Figure 3C).

### 3.4. Serum Biochemical Parameters in GBS Patients During Recovery

**Figure 4:**
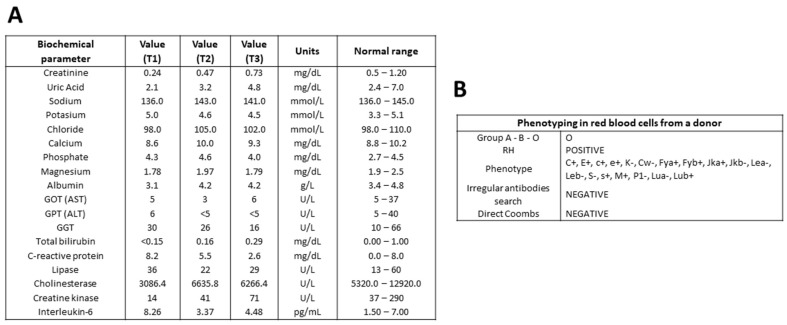
**Representative determination of serum biochemical parameters from a patient with GBS and phenotyping of red blood cells from a control donor.** Serum analysis of various biochemical parameters at time points T1, T2, and T3 during the recovery of a male patient with GBS (**A**). Phenotyping of red blood cells obtained from the blood of a male volunteer donor (**B**). AST, serum glutamic–oxaloacetic transaminase or aspartate aminotransferase; ALT, serum glutamic–pyruvic transaminase or alanine aminotransferase; GGT, gamma–glutamyl transferase.

Eighteen serum biochemical parameters from patients with GBS and controls are being studied in the GBS Biobank. Samples from each GBS patient, corresponding to different stages of the disease, are analyzed simultaneously to minimize variability in measurements. In a representative determination, values of biochemical parameters, such as creatinine, uric acid, calcium, albumin, bilirubin, C-reactive protein, cholinesterase, creatine kinase, and IL-6, tended toward the normal range. Some parameters remained within the normal range at different stages of GBS: sodium, potassium, chloride, phosphate, gamma-glutamyl transferase, and lipase. Liver transferases, GOT, GPT, and magnesium showed fluctuations within the lower limit of the accepted range (Figure 4A).

### 3.5. Phenotyping of Red Blood Cells in Donors of GBS Biobank Collection

Red blood cell phenotyping is performed on the EDTA-anticoagulated blood of each GBS patient, tSCI patient, or control donor on the same day as the first blood draw. ABO blood type and RH are determined, as well as a panel of several surface antigens. A search for irregular antibodies and a direct Coombs’ test complete the blood antigen analysis. An example of individual phenotype screening is shown in Figure 4B.

## 4. Discussion

Guillain–Barré syndrome (GBS) remains the most common cause of acute neuromuscular paralysis globally and can pose a life-threatening risk, underscoring the importance of early intervention to improve outcomes. Although considerable progress has been made in elucidating its post-infectious, immune-mediated pathophysiology, key aspects of the disease remain incompletely understood. Diagnosis relies primarily on specialized clinical evaluation by experienced clinicians, while therapeutic options are still largely confined to intravenous immunoglobulin or plasma exchange [1,16,25].

The Hospital Nacional de Parapléjicos (HNP) in Toledo serves as a national referral center for Guillain–Barré syndrome (GBS) in Spain, receiving patients from across the country. Patients are typically transferred to the HNP following an initial diagnosis and insufficient response to treatment at a general hospital. Admission to the HNP under these circumstances often requires additional clinical criteria, such as respiratory failure requiring mechanical ventilation or failure to recover from persistent motor weakness beyond four weeks, indicative of a prolonged nadir. In both scenarios, initial onset and prolonged nadir, patients undergo repeated diagnostic evaluation, resulting in a confirmed GBS diagnosis rate exceeding 95%, even in the absence of electromyography upon arrival at HNP.

For the BioGBS study, blood samples were collected from each GBS patient at the HNP at defined time points during recovery (T1, T2, and T3); samples at the onset of GBS (T0) were obtained from the local general hospital. Patients with traumatic spinal cord injury (tSCI) at the HNP and healthy individuals sampled at the regional blood bank were included as control groups. Donor whole blood collected at the HNP, the local general hospital, and the blood bank were processed by biobank staff to avoid blood processing bias and comply with safety regulations. PBMCs and serum samples isolated from whole blood from three groups comprise the GBS Biobank Collection at HNP.

Integrating GBS Biobank Collection as resource into our BioGBS studies on gene expression, proteomic analysis, blood biochemical parameters, or phenotyping offers several advantages. One of the main advantages of using a biobank is access to high-quality, well-preserved biological samples under standardized conditions, which ensures sample integrity and reduces variability in DNA, RNA, protein, and biochemical measurements. This consistency is crucial for reliable biomarker analysis, especially when investigating gene and protein expression or biochemical changes in patients and controls [26].

Furthermore, biobanks often collect longitudinal samples, allowing for monitoring changes in biomolecules and enzyme activity over time [27,28]. This feature is particularly valuable for the study of GBS, as it allows for the evaluation of genes, proteins, or biochemical fluctuations at different stages of the disease. By analyzing serial samples from same GBS patient, we can examine possible patterns in the progression of GBS and their relationship with the blood biomolecular markers described [29]; see Figure 1 and Figure 2.

Another advantage is the availability of control samples, including samples from healthy individuals or tSCI patients with central or peripheral neuropathies described in tSCI pathology, such as neuropathic pain or nerve root injuries [30,31]. These controls are essential for distinguishing GBS-specific biomarkers from those associated with other conditions. Furthermore, some biobanks integrate clinical records with biological samples, providing valuable datasets for studying biochemical variations in different patient populations [32].

Thanks to multicenter collaboration, the GBS Biobank collection allowed us to track clinical data from the onset, acute phase, and early-to-moderate recovery of GBS patients, comparing them with controls. To study the progression and recovery of GBS, different time points from the same patient are appropriate (see Figure 4); to distinguish the specificity of the neurodegenerative disease type, healthy and SCI groups are useful for comparison [33].

The International GBS Outcomes Study (IGOS) is a global prognostic study that has been collecting information on the immune-related pathophysiology and outcomes of GBS in both children and adults [34,35]. This is a large consortium where more than 1,400 patients meeting the diagnostic criteria for GBS were eligible for inclusion, regardless of age, disease severity, variant, or treatment, provided they enrolled within two weeks of onset of weakness. Data on demographics, previous infections, clinical features, diagnostic findings, treatment, disease progression, and outcomes are collected, along with cerebrospinal fluid and DNA samples at GBS onset, as well as serial blood samples for serum at predefined time points until 52 weeks [36].

The BioGBS study is currently being conducted in a cohort of 64 patients with acute Guillain–Barré syndrome lasting more than four weeks who were unresponsive to treatment and underwent serial in-hospital serum and PBMC analysis from defined recovery time points until discharge. More recently, the study has expanded to include patients enrolled at the onset of weakness. This allows for a comparative analysis of therapeutic response and disease progression between early-stage and treatment-resistant cases.

**Figure 5:**
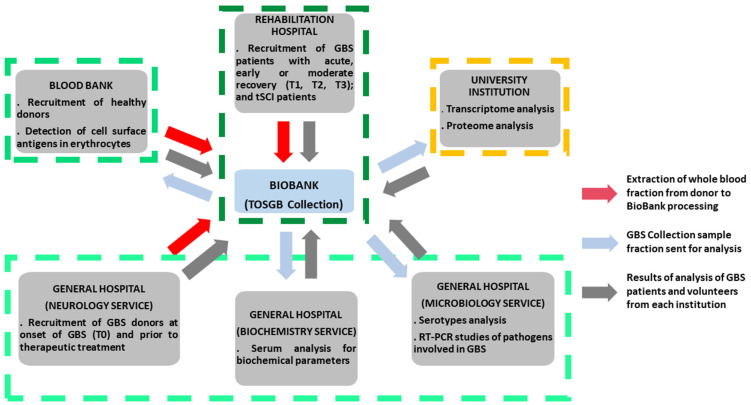
**Central role of GBS Biobank Collection (TOSGB) for intra-hospital and inter-institutional interaction in BioGBS study**.

The Biobank’s GBS collection is platform for BioGBS project at HNP. It plays a critical role in coordinating collaborations across different medical and academic institutions. This ranges from the recruitment and inpatient follow-up of GBS patients in rehabilitation hospitals to interaction with neurology, biochemistry, and microbiology departments, blood banks for phenotyping and recruitment of healthy donors, and even with research institutes specializing in transcriptomic and proteomic analysis. The availability of well-characterized blood samples facilitates comprehensive biochemical investigations, including enzyme activity assays, metabolic profiles, and immunological assessments. In microbiology departments, biobank resources enable the study of infectious agents or microbial interactions that may influence disease onset and progression in GBS patients (Figure 5).

Beyond biochemical analysis, biobank resources may provide access to genetic and proteomic data, offering further insights into the molecular mechanisms of GBS. Genetic predispositions, inflammatory pathways, and proteomic changes associated with disease progression could be explored using these datasets, thereby broadening the scope of our research [37,38].

PBMCs samples provide valuable information on the cellular immune response in GBS, enabling transcriptomic, proteomic, and functional analyses over the course of the disease [39,40]. However, many soluble biomarkers—such as cytokines, complement proteins, and neurofilament light chain—are more reliably detected in plasma or serum, each offering distinct advantages depending on the analyte [41,42]. Therefore, a comprehensive GBS biobank should include both plasma and serum in addition to PBMCs to support broad biomarker discovery and validation. Furthermore, cerebrospinal fluid (CSF) is critical for identifying markers of nerve damage, inflammation, and blood–nerve barrier dysfunction that may not be present in peripheral blood [43,44]. Including PBMCs, plasma, serum, and CSF allows for a more complete characterization of the immunological and pathophysiological processes involved in GBS.

In this study, plasma and CSF were not included in the GBS Biobank Collection due to logistical and practical limitations. Plasma collection requires careful standardization of anticoagulant use and immediate processing to avoid degradation of sensitive biomarkers, which was not feasible under the routine clinical conditions of the participating centers [45,46]. Similarly, CSF collection is an invasive procedure that depends on clinical indication and patient consent, and is not consistently performed across all patients, particularly those with milder disease [13,47]. These constraints limited the ability to systematically include plasma and CSF samples, leading this study to focus primarily on PBMCs and serum, which are more readily obtainable within the clinical workflow.

Finally, leveraging biobank resources enhances the potential for multi-center collaborations, increasing the generalizability of findings. A broader and more diverse sample set improves the robustness of statistical analyses, strengthening the validity of identified biomarkers. Future studies should consider expanding the use of biobank data to include a wider range of biochemical and molecular analyses to further elucidate the complexity of pathophysiology pathways related to GBS.

The use of samples from Guillain–Barré syndrome Biobank Collections, while invaluable for advancing research on this rare and heterogeneous disease, has some limitations. One major limitation is the small sample size, as the low incidence of GBS makes it difficult to collect large and statistically robust cohorts. Furthermore, the clinical heterogeneity of GBS, ranging from different subtypes (AIDP, AMAN, AMSAN, and MFS) to varying disease severity and treatment regimens, can introduce variability that makes data interpretation difficult. For biobank samples, the lack of comprehensive longitudinal clinical data limits the ability to assess disease progression or long-term outcomes. Furthermore, it is crucial to reduce differences in sample collection, processing, and storage protocols that could affect sample quality and comparability. Ethical and legal considerations, such as restrictions on data sharing and renewing patient consent for new research purposes, can also hinder the wider use of these valuable resources. These limitations must be carefully addressed when designing studies to ensure the validity and reproducibility of findings derived from GBS biobank samples.

## 5. Conclusions

In this article, we describe studies related to the BioGBS project, including the current cohort of GBS patients and controls, as well as the characteristics of each donor group. We present the blood collection procedures, processing, and storage of human serum and PBMC samples for the GBS Biobank collection, as well as the sampling time points during the course of GBS. We summarize the analysis of GBS Biobank samples using transcriptomics and proteomics, which provided specific biomarkers for GBS. Current results on serum biochemical parameters and phenotyping were presented. Finally, we propose a central role for the GBS Biobank collection in the BioGBS project, integrating all research from different healthcare or academic institutions, which is especially useful in rare disease research.

## Figures and Tables

**Table 1:** Methodology for determining biochemical and ionic parameters.

Biochemical Parameter	Detection Method
Creatinine	Jaffé reaction
Uric Acid	Uricase-Peroxidase; enzymatic colorimetric
Sodium	Indirect potentiometry
Potassium	Indirect potentiometry
Chloride	Indirect potentiometry
Calcium	Photometric NM-BAPTA/EDTA determination
Phosphate	Photometric molybdate
Magnesium	Xylidyl blue colorimetric method (end-point determination)
Albumin	Immunoturbidimetry
AST/GOT (Aspartate Aminotransferase)	Enzymatic UV method (NADH-linked)
ALT/GPT (Alanine Aminotransferase)	Enzymatic UV Method (NADH-linked)
GGT (Gamma Glutamyl Transferase)	Enzymatic Colorimetric Method with γ-Glutamyl-3-carboxy-4-nitroanilide substrate
Total Bilirubin	Colorimetric Diazo method
C-reactive Protein	Latex particles-enhanced immunoturbidimetry
Lipase	Colorimetric enzymatic method by chromogenic substrate (1,2-O-dilauryl-rac-glycero-3-glutaric acid-(6′-methylresorufin) ester)
Cholinesterase	Colorimetric assay using butyrylthiocholine and hexacyanoferrate
Creatine Kinase	UV kinetic enzymatic method (NADPH-linked)
Interleukin-6	Electrochimioluminiscense immunoessay

## Data Availability

Data are contained within the article and Supplementary Materials. The data of biological samples of human origin from TOSGB Collection are coding for donor anonymization. The TOSGB Collection belongs to the Biobank of Hospital Universitario de Toledo, see the relevant permissions and original reports in the Supplementary Materials.

## Supplementary Materials

The following supporting information can be downloaded at: https://www.mdpi.com/article/10.3390/diagnostics15151900/s1, Figure S1: Approval by the Toledo Clinical Research Ethics Committee (2014) and the Clinical Research Commission of the Paraplegic Hospital (2022) for the BioGBS project; Figure S2: Approval of the TOSGB Biobank Collection by the Toledo Research Commission (February 2018) and the Counselor of Health of the Regional Government of Castilla-La Mancha (November 2018); Figure S3: Official document (June 2017) certifying that the biological samples of human origin for biomedical research purposes from the TOSGB Collection are part of the Biobank of Hospital Universitario de Toledo (formerly BioB-HVS); it includes sample coding for donor anonymization; Figure S4: Original reports on the determination of serum biochemical parameters of a patient with GBS at recovery points T1, T2 and T3 at the National Paraplegic Hospital; Figure S5: Original report on red blood cell phenotyping in a whole blood sample from a man donor included in the BioGBS study.
