# Supplementary material for: Hospital Coordination and Protocols Using Serum and Peripheral Blood Cells from Patients and Healthy Donors in a Longitudinal Study of Guillain–Barré Syndrome
Source: Diagnostics (Basel). 2025 Jul 29;15(15):1900. doi: 10.3390/diagnostics15151900 (PMC12346330; doi:10.3390/diagnostics15151900)
Supplement: Supplementary file 1 [file diagnostics-15-01900-s001.zip › diagnostics-3668549-supplementary.pdf]

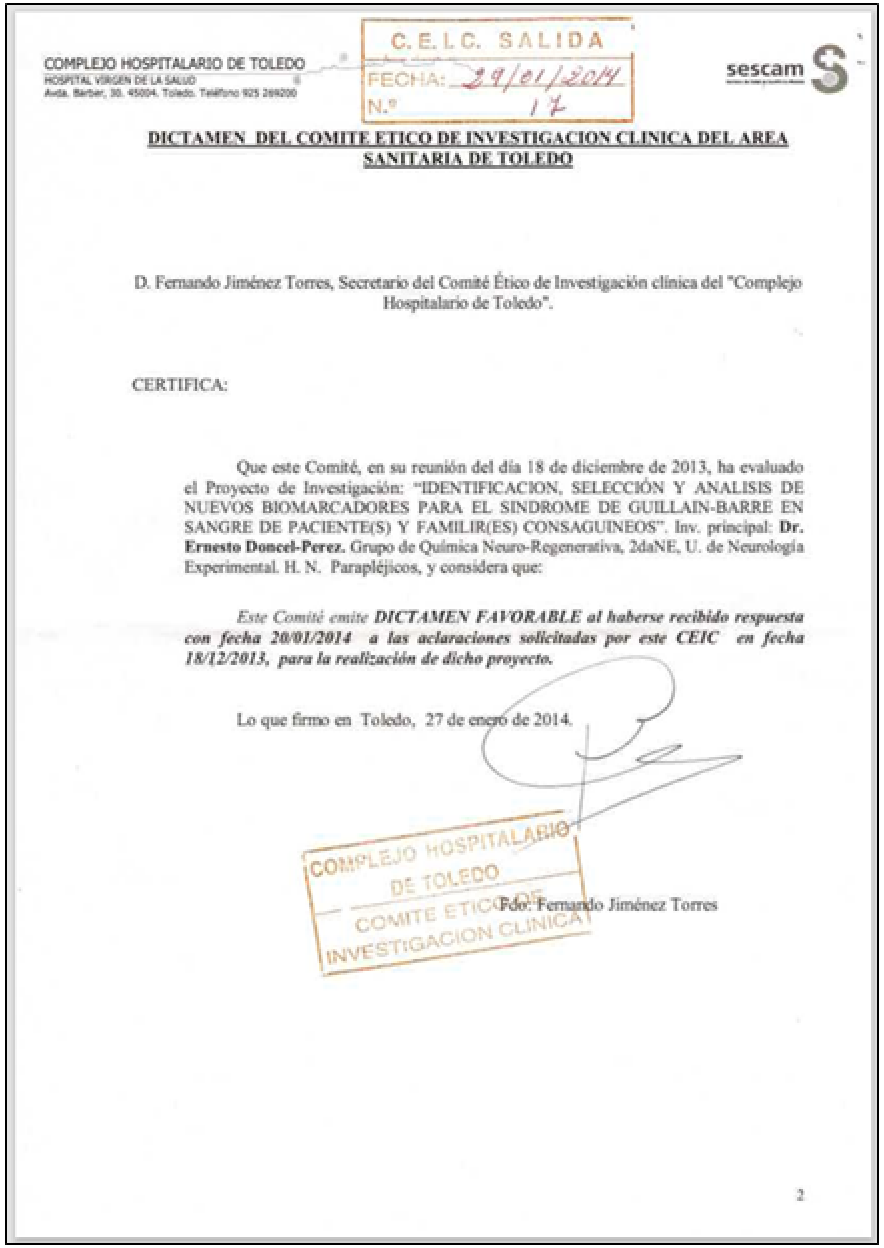

COMPLEJO HOSPITALARIO DE TOLEDO
HOSPITAL VIRGEN DE LA SALUD
Avda. Barber, 30. 45004. Toledo. Teléfono 925 269200

C.E.I.C. SALIDA
FECHA: 29/01/2014
N.º 17

sescam

DICTAMEN DEL COMITE ETICO DE INVESTIGACION CLINICA DEL AREA SANITARIA DE TOLEDO

D. Fernando Jiménez Torres, Secretario del Comité Ético de Investigación clínica del "Complejo Hospitalario de Toledo".

CERTIFICA:

Que este Comité, en su reunión del día 18 de diciembre de 2013, ha evaluado el Proyecto de Investigación: "IDENTIFICACION, SELECCIÓN Y ANALISIS DE NUEVOS BIOMARCADORES PARA EL SINDROME DE GUILLAIN-BARRE EN SANGRE DE PACIENTE(S) Y FAMILIR(ES) CONSAGUINEOS". Inv. principal: **Dr. Ernesto Doncel-Perez.** Grupo de Química Neuro-Regenerativa, 2daNE, U. de Neurología Experimental. H. N. Parapléjicos, y considera que:

Este Comité emite ***DICTAMEN FAVORABLE al haberse recibido respuesta con fecha 20/01/2014 a las aclaraciones solicitadas por este CEIC en fecha 18/12/2013, para la realización de dicho proyecto.***

Lo que firmo en Toledo, 27 de enero de 2014.

COMPLEJO HOSPITALARIO DE TOLEDO
COMITE ETICO DE INVESTIGACION CLINICA

Fdo: Fernando Jiménez Torres

2

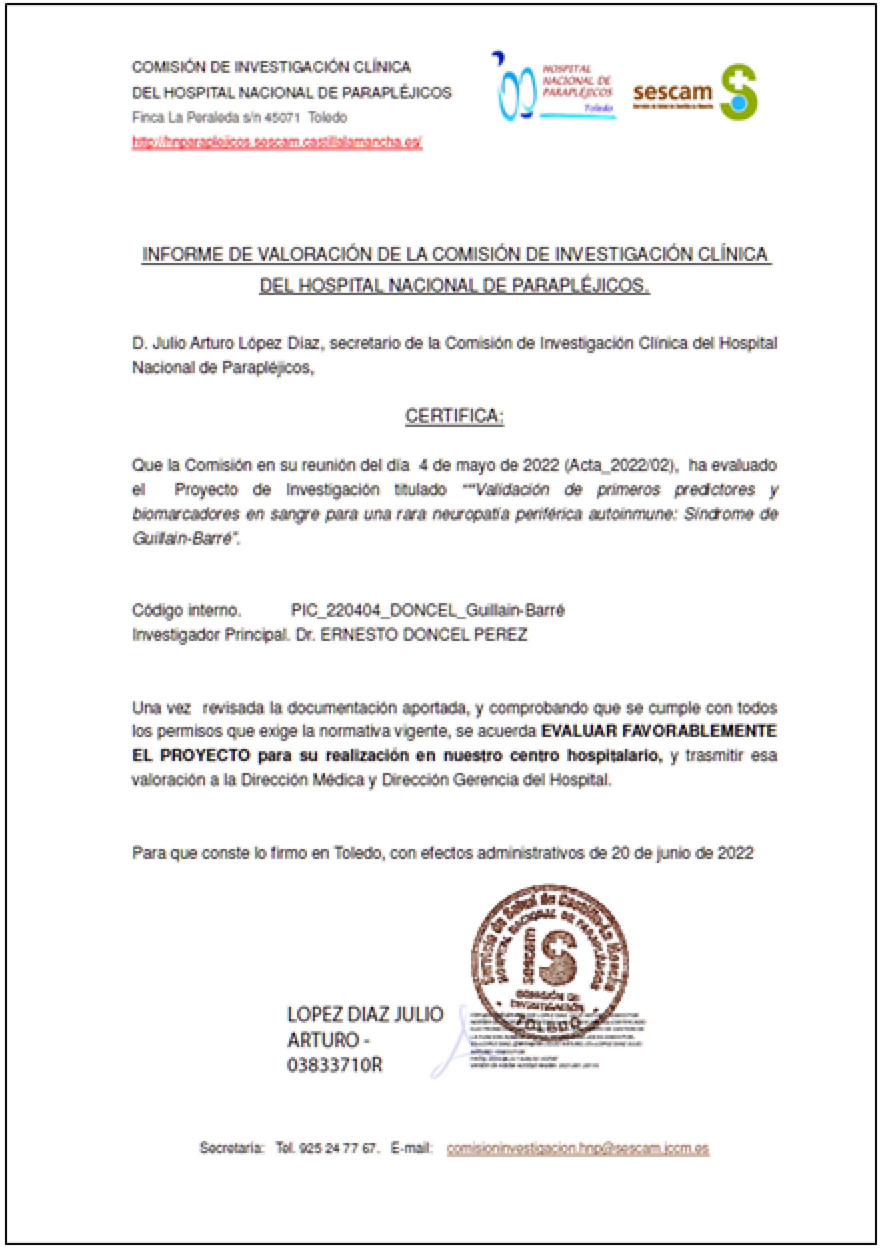

COMISIÓN DE INVESTIGACIÓN CLÍNICA
DEL HOSPITAL NACIONAL DE PARAPLÉJICOS
Finca La Peraleda s/n 45071 Toledo
http://hnparaplejicos.sescam.castillalamancha.es/

HOSPITAL NACIONAL DE PARAPLÉJICOS Toledo
sescam

INFORME DE VALORACIÓN DE LA COMISIÓN DE INVESTIGACIÓN CLÍNICA DEL HOSPITAL NACIONAL DE PARAPLÉJICOS.

D. Julio Arturo López Díaz, secretario de la Comisión de Investigación Clínica del Hospital Nacional de Parapléjicos,

CERTIFICA:

Que la Comisión en su reunión del día 4 de mayo de 2022 (Acta_2022/02), ha evaluado el Proyecto de Investigación titulado ""*Validación de primeros predictores y biomarcadores en sangre para una rara neuropatía periférica autoinmune: Síndrome de Guillain-Barré*".

Código interno. PIC_220404_DONCEL_Guillain-Barré
Investigador Principal. Dr. ERNESTO DONCEL PEREZ

Una vez revisada la documentación aportada, y comprobando que se cumple con todos los permisos que exige la normativa vigente, se acuerda **EVALUAR FAVORABLEMENTE EL PROYECTO para su realización en nuestro centro hospitalario,** y trasmitir esa valoración a la Dirección Médica y Dirección Gerencia del Hospital.

Para que conste lo firmo en Toledo, con efectos administrativos de 20 de junio de 2022

LOPEZ DIAZ JULIO ARTURO - 03833710R

Secretaría: Tel. 925 24 77 67. E-mail: comisioninvestigacion.hnp@sescam.jccm.es

Figure S1. Approval by the Toledo Clinical Research Ethics Committee (2014) and the Clinical Research Commission of the Paraplegic Hospital (2022) for the BioGBS project.

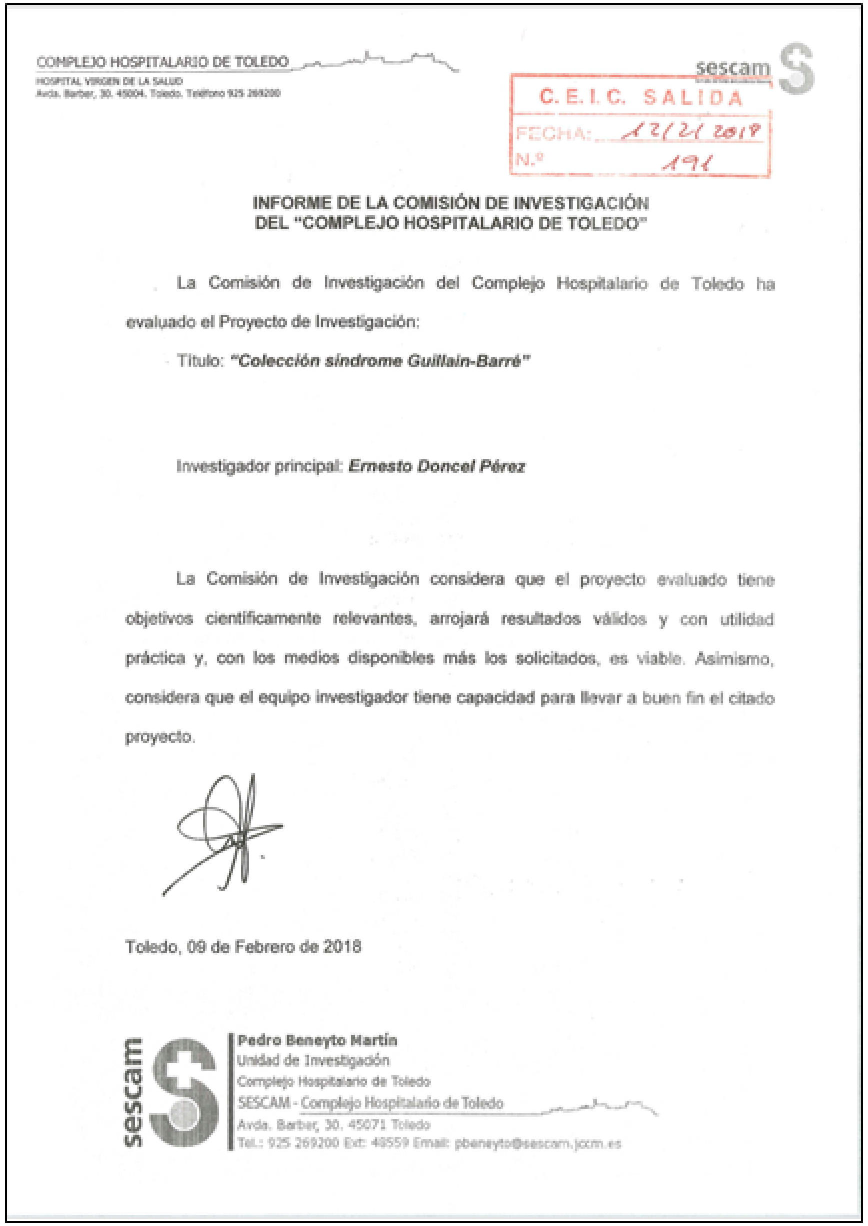

COMPLEJO HOSPITALARIO DE TOLEDO
HOSPITAL VIRGEN DE LA SALUD
Avda. Barber, 30. 45004. Toledo. Teléfono 925 269200

sescam

C.E.I.C. SALIDA
FECHA: 12/2/2018
N.º 191

INFORME DE LA COMISIÓN DE INVESTIGACIÓN DEL "COMPLEJO HOSPITALARIO DE TOLEDO"

La Comisión de Investigación del Complejo Hospitalario de Toledo ha evaluado el Proyecto de Investigación:

Título: ***"Colección síndrome Guillain-Barré"***

Investigador principal: ***Ernesto Doncel Pérez***

La Comisión de Investigación considera que el proyecto evaluado tiene objetivos científicamente relevantes, arrojará resultados válidos y con utilidad práctica y, con los medios disponibles más los solicitados, es viable. Asimismo, considera que el equipo investigador tiene capacidad para llevar a buen fin el citado proyecto.

Toledo, 09 de Febrero de 2018

sescam
Pedro Beneyto Martín
Unidad de Investigación
Complejo Hospitalario de Toledo
SESCAM - Complejo Hospitalario de Toledo
Avda. Barber, 30. 45071 Toledo
Tel.: 925 269200 Ext: 49559 Email: pbeneyto@sescam.jccm.es

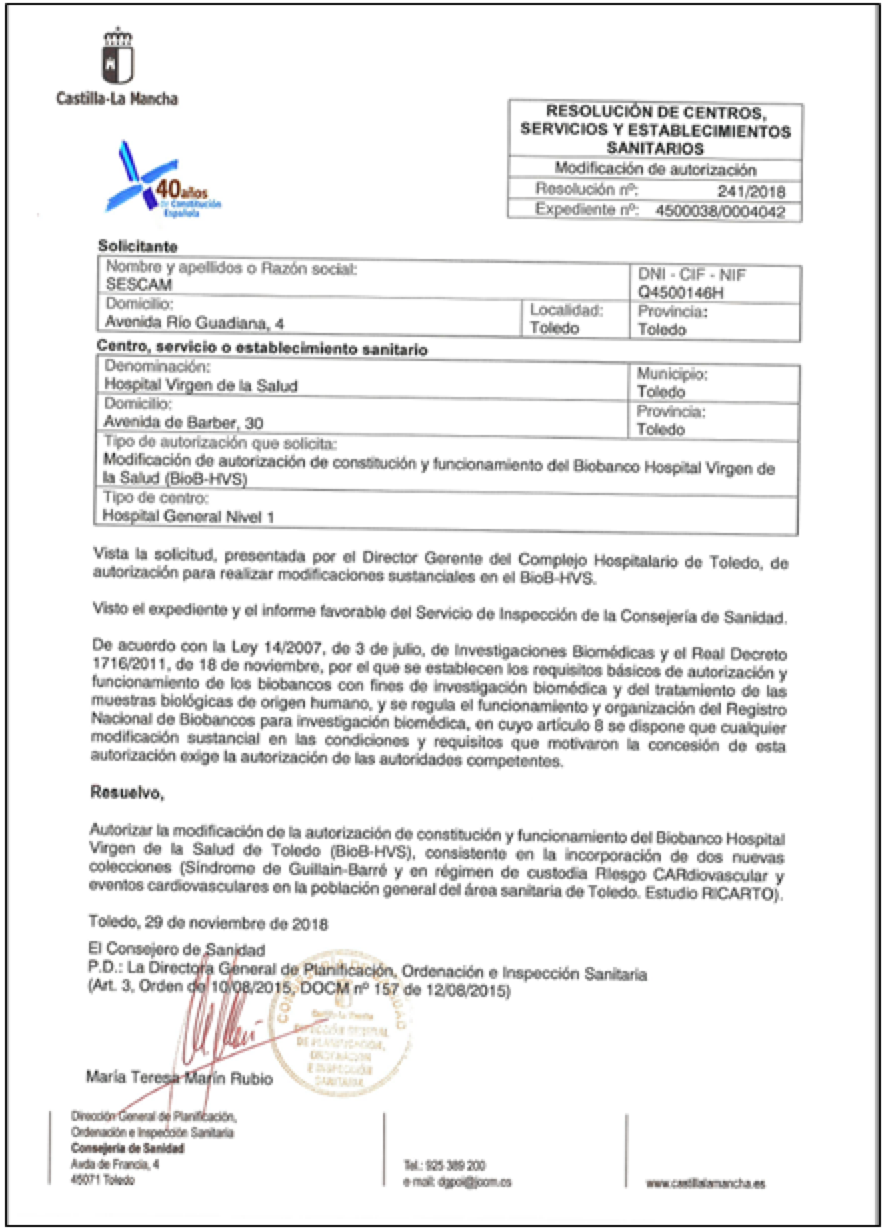

Castilla-La Mancha

40 años de Constitución Española

RESOLUCIÓN DE CENTROS, SERVICIOS Y ESTABLECIMIENTOS SANITARIOS	
Modificación de autorización	
Resolución nº:	241/2018
Expediente nº:	4500038/0004042

Solicitante

Nombre y apellidos o Razón social: SESCAM		DNI - CIF - NIF Q4500146H
Domicilio: Avenida Río Guadiana, 4	Localidad: Toledo	Provincia: Toledo

Centro, servicio o establecimiento sanitario

Denominación: Hospital Virgen de la Salud	Municipio: Toledo
Domicilio: Avenida de Barber, 30	Provincia: Toledo
Tipo de autorización que solicita: Modificación de autorización de constitución y funcionamiento del Biobanco Hospital Virgen de la Salud (BioB-HVS)	
Tipo de centro: Hospital General Nivel 1	

Vista la solicitud, presentada por el Director Gerente del Complejo Hospitalario de Toledo, de autorización para realizar modificaciones sustanciales en el BioB-HVS.

Visto el expediente y el informe favorable del Servicio de Inspección de la Consejería de Sanidad.

De acuerdo con la Ley 14/2007, de 3 de julio, de Investigaciones Biomédicas y el Real Decreto 1716/2011, de 18 de noviembre, por el que se establecen los requisitos básicos de autorización y funcionamiento de los biobancos con fines de investigación biomédica y del tratamiento de las muestras biológicas de origen humano, y se regula el funcionamiento y organización del Registro Nacional de Biobancos para investigación biomédica, en cuyo artículo 8 se dispone que cualquier modificación sustancial en las condiciones y requisitos que motivaron la concesión de esta autorización exige la autorización de las autoridades competentes.

Resuelvo,

Autorizar la modificación de la autorización de constitución y funcionamiento del Biobanco Hospital Virgen de la Salud de Toledo (BioB-HVS), consistente en la incorporación de dos nuevas colecciones (Síndrome de Guillain-Barré y en régimen de custodia RIesgo CARdiovascular y eventos cardiovasculares en la población general del área sanitaria de Toledo. Estudio RICARTO).

Toledo, 29 de noviembre de 2018

El Consejero de Sanidad
P.D.: La Directora General de Planificación, Ordenación e Inspección Sanitaria
(Art. 3, Orden de 10/08/2015, DOCM nº 157 de 12/08/2015)

María Teresa Marín Rubio

Dirección General de Planificación, Ordenación e Inspección Sanitaria
Consejería de Sanidad
Avda de Francia, 4
45071 Toledo

Tel.: 925 389 200
e-mail: dgpoi@jccm.es

www.castillalamancha.es

Figure S2. Approval of the TOSGB Biobank Collection by the Toledo Research Commission (February 2018) and the Counselor of Health of the Regional Government of Castilla-La Mancha (November 2018).

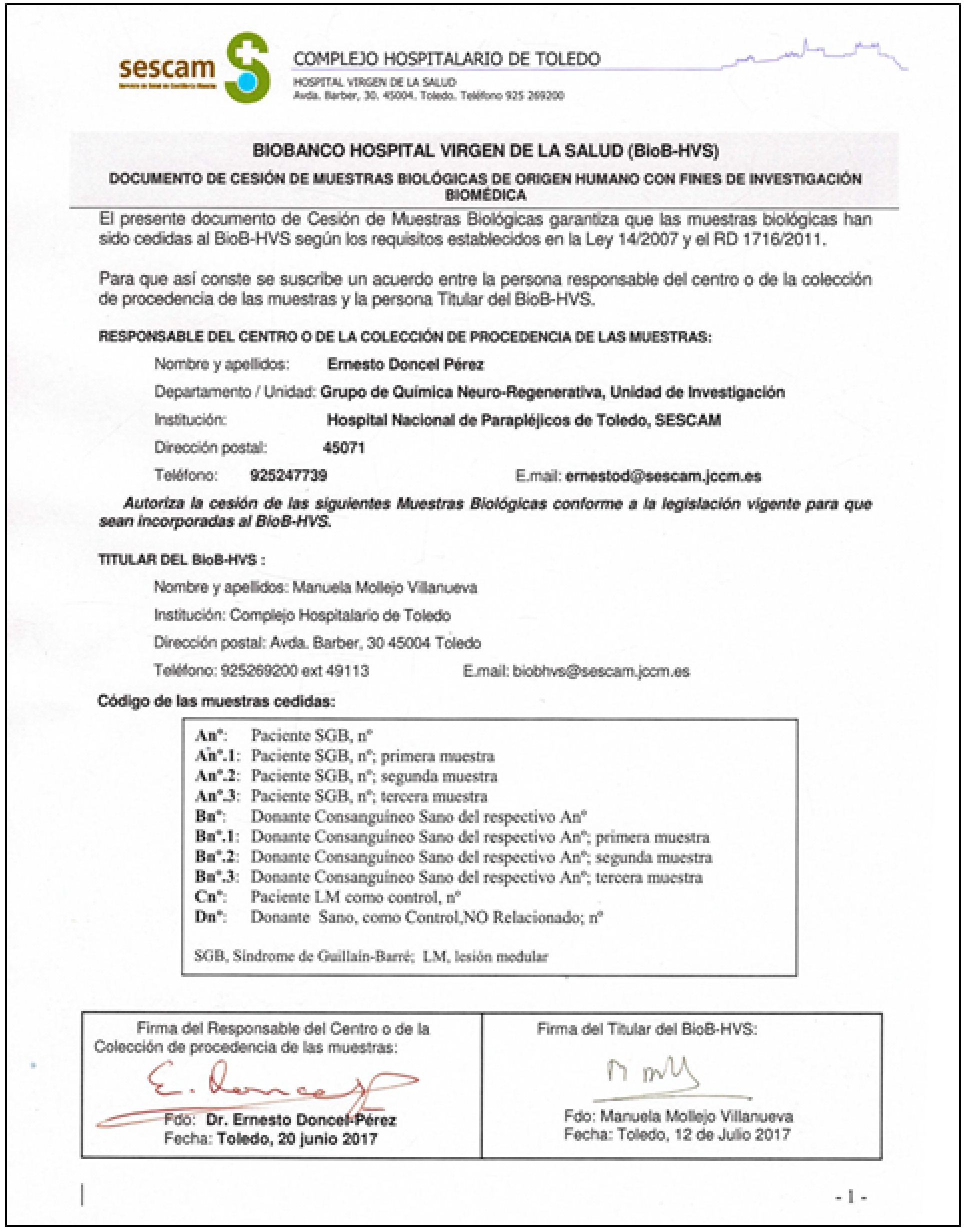

sescam

COMPLEJO HOSPITALARIO DE TOLEDO

HOSPITAL VIRGEN DE LA SALUD

Avda. Barber, 30. 45004. Toledo. Teléfono 925 269200

BIOBANCO HOSPITAL VIRGEN DE LA SALUD (BioB-HVS)

DOCUMENTO DE CESIÓN DE MUESTRAS BIOLÓGICAS DE ORIGEN HUMANO CON FINES DE INVESTIGACIÓN BIOMÉDICA

El presente documento de Cesión de Muestras Biológicas garantiza que las muestras biológicas han sido cedidas al BioB-HVS según los requisitos establecidos en la Ley 14/2007 y el RD 1716/2011.

Para que así conste se suscribe un acuerdo entre la persona responsable del centro o de la colección de procedencia de las muestras y la persona Titular del BioB-HVS.

RESPONSABLE DEL CENTRO O DE LA COLECCIÓN DE PROCEDENCIA DE LAS MUESTRAS:

Nombre y apellidos: **Ernesto Doncel Pérez**

Departamento / Unidad: **Grupo de Química Neuro-Regenerativa, Unidad de Investigación**

Institución: **Hospital Nacional de Parapléjicos de Toledo, SESCAM**

Dirección postal: **45071**

Teléfono: **925247739** E.mail: **ernestod@sescam.jccm.es**

Autoriza la cesión de las siguientes Muestras Biológicas conforme a la legislación vigente para que sean incorporadas al BioB-HVS.

TITULAR DEL BioB-HVS :

Nombre y apellidos: Manuela Mollejo Villanueva

Institución: Complejo Hospitalario de Toledo

Dirección postal: Avda. Barber, 30 45004 Toledo

Teléfono: 925269200 ext 49113 E.mail: biobhvs@sescam.jccm.es

Código de las muestras cedidas:

**Anº:**	Paciente SGB, nº
**Anº.1:**	Paciente SGB, nº; primera muestra
**Anº.2:**	Paciente SGB, nº; segunda muestra
**Anº.3:**	Paciente SGB, nº; tercera muestra
**Bnº:**	Donante Consanguíneo Sano del respectivo Anº
**Bnº.1:**	Donante Consanguíneo Sano del respectivo Anº; primera muestra
**Bnº.2:**	Donante Consanguíneo Sano del respectivo Anº; segunda muestra
**Bnº.3:**	Donante Consanguíneo Sano del respectivo Anº; tercera muestra
**Cnº:**	Paciente LM como control, nº
**Dnº:**	Donante Sano, como Control,NO Relacionado; nº

SGB, Síndrome de Guillain-Barré; LM, lesión medular

Firma del Responsable del Centro o de la Colección de procedencia de las muestras:	Firma del Titular del BioB-HVS:
Fdo: **Dr. Ernesto Doncel-Pérez**<br>Fecha: **Toledo, 20 junio 2017**	Fdo: Manuela Mollejo Villanueva<br>Fecha: Toledo, 12 de Julio 2017

- 1 -

Figure S3. Official document (June 2017) certifying that the biological samples of human origin for biomedical research purposes from the TOSGB Collection are part of the Biobank of Hospital Universitario de Toledo (formerly BioB-HVS); it includes sample coding for donor anonymization.

Paciente: **MOMENTO1, A10**
NHC CHUT: **A10.Momento1** NHC HNP:
Servicio: NO CONSTA - DESCONOCIDO
Peticionario: No consta
Centro: Hospital U. Toledo

Número: **00005848**
Edad: 47 años Sexo: M
Urgente: **No**
Fecha análisis: 23/5/24
Cierre informe: 26/5/24 15:45:45

Copia de Laboratorio

LABORATORIO DE BIOQUIMICA

BIOQUIMICA EN SANGRE

Resultados validados por: Dra. María Zurdo López (Residente)

Creatinina		**0.24**	mg/dL	0.50 - 1.20
Ac. Úrico		**2.1**	mg/dL	2.4 - 7.0
Sodio		**136.0**	mmol/L	136.0 - 145.0
Potasio		**5.0**	mmol/L	3.3 - 5.1
Cloro		**98.0**	mmol/L	98.0 - 110.0
Calcio		**8.6**	mg/dL	8.8 - 10.2
Fósforo		**4.3**	mg/dL	2.7 - 4.5
Magnesio	*	**1.78**	mg/dL	1.9 - 2.5
Albúmina		**3.1**	g/dL	3.4 - 4.8
GOT(AST)		**5**	U/L	5 - 37
GPT(ALT)		**6**	U/L	5 - 40
GGT		**30**	U/L	10 - 66
Bilirrubina Total		**<0.15**	mg/dL	0.00 - 1.00
Proteina C reactiva	*	**8.2**	mg/L	0.0 - 8.0
Lipasa		**36**	U/L	13- 60
Colinesterasa		**3086.4**	U/L	5320.0 - 12920.0
CK	**	**14**	U/L	37 - 290
Interleucina-6	*	**8.26**	pg/mL	1.50- 7.00

La determinación de IL-6 está indicada antes de iniciar el tratamiento con Tocilizumab (niveles mayores de 40 pg/ml en adultos y de 35 pg/ml en niños). Su utilidad clínica en la monitorización del tratamiento es muy escasa, ya que suele producirse un incremento en los niveles de IL-6.

Paciente: **MOMENTO2, A10**
NHC CHUT: **A10.Momento2** NHC HNP:
Servicio: NO CONSTA - DESCONOCIDO
Peticionario: No consta
Centro: Hospital U. Toledo

Número: **00005849**
Edad: 47 años Sexo: M
Urgente: **No**
Fecha análisis: 23/5/24
Cierre informe: 27/5/24 13:59:52

Copia de Laboratorio

LABORATORIO DE BIOQUIMICA

BIOQUIMICA EN SANGRE

Resultados validados por: Dra. Ana Cristina Fernández Castro, Dr. Juan José Palomar Pérez

Creatinina	**0.47**	mg/dL	0.50 - 1.20
Ac. Úrico	**3.2**	mg/dL	2.4 - 7.0
Sodio	**143.0**	mmol/L	136.0 - 145.0
Potasio	**4.6**	mmol/L	3.3 - 5.1
Cloro	**105.0**	mmol/L	98.0 - 110.0
Calcio	**10.0**	mg/dL	8.8 - 10.2
Fósforo	**4.6**	mg/dL	2.7 - 4.5
Magnesio	**1.97**	mg/dL	1.9 - 2.5
Albúmina	**4.2**	g/dL	3.4 - 4.8
GOT(AST)	**3**	U/L	5 - 37
GPT(ALT)	**<5**	U/L	5 - 40
GGT	**26**	U/L	10 - 66
Bilirrubina Total	**0.16**	mg/dL	0.00 - 1.00
Proteina C reactiva	**5.5**	mg/L	0.0 - 8.0
Lipasa	**22**	U/L	13- 60
Colinesterasa	**6635.8**	U/L	5320.0 - 12920.0
CK	**41**	U/L	37 - 290
Interleucina-6	**3.37**	pg/mL	1.50- 7.00

La determinación de IL-6 está indicada antes de iniciar el tratamiento con Tocilizumab (niveles mayores de 40 pg/ml en adultos y de 35 pg/ml en niños). Su utilidad clínica en la monitorización del tratamiento es muy escasa, ya que suele producirse un incremento en los niveles de IL-6.

Paciente: **MOMENTO3, A10**
NHC CHUT: **A10.Momento3** NHC HNP:
Servicio: NO CONSTA - DESCONOCIDO
Peticionario: No consta
Centro: Hospital U. Toledo

Número: **00005851**
Edad: 47 años Sexo: M
Urgente: **No**
Fecha análisis: 23/5/24
Cierre informe: 26/5/24 15:45:59

Copia de Laboratorio

LABORATORIO DE BIOQUIMICA

BIOQUIMICA EN SANGRE

Resultados validados por: Dra. María Zurdo López (Residente)

Creatinina		**0.73**	mg/dL	0.50 - 1.20
Ac. Úrico		**4.8**	mg/dL	2.4 - 7.0
Sodio		**141.0**	mmol/L	136.0 - 145.0
Potasio		**4.5**	mmol/L	3.3 - 5.1
Cloro		**102.0**	mmol/L	98.0 - 110.0
Calcio		**9.3**	mg/dL	8.8 - 10.2
Fósforo		**4.0**	mg/dL	2.7 - 4.5
Magnesio	*	**1.79**	mg/dL	1.9 - 2.5
Albúmina		**4.2**	g/dL	3.4 - 4.8
GOT(AST)		**6**	U/L	5 - 37
GPT(ALT)		**<5**	U/L	5 - 40
GGT		**16**	U/L	10 - 66
Bilirrubina Total		**0.29**	mg/dL	0.00 - 1.00
Proteina C reactiva		**2.6**	mg/L	0.0 - 8.0
Lipasa		**29**	U/L	13- 60
Colinesterasa		**6266.4**	U/L	5320.0 - 12920.0
CK		**71**	U/L	37 - 290
Interleucina-6		**4.48**	pg/mL	1.50- 7.00

La determinación de IL-6 está indicada antes de iniciar el tratamiento con Tocilizumab (niveles mayores de 40 pg/ml en adultos y de 35 pg/ml en niños). Su utilidad clínica en la monitorización del tratamiento es muy escasa, ya que suele producirse un incremento en los niveles de IL-6.

Figure S4. Original reports on the determination of serum biochemical parameters of a patient with GBS at recovery points T1, T2 and T3 at the National Paraplegic Hospital.

CENTRO REGIONAL DE TRANSFUSIÓN
TOLEDO-GUADALAJARA
HOSPITAL VIRGEN DE LA SALUD
Avda. Barber, 30. 45004. Teléfono 925 269 200
Toledo.

sescam

Paciente: [redacted] F.Nac: [redacted] Sexo: HOMBRE
Historia Clínica: Tarjeta sanitaria: Seguridad Social:
Médico:
Origen: HOSPITAL NACIONAL PARAPLEJICOS
Servicio: HOSPITAL NACIONAL PARAPLEJICOS
Ubicación:

Muestra número: 000056 (26/02/2025)

RESULTADOS ANALÍTICOS

Grupo A - B - O	O
RH	POSITIVO
Fenotipos	C+, E-, c+, e+, K-, Fya+, Fyb+, Jka+, Jkb-, Lea-, Leb+, S+, s+, M+, P1+, Lua-, Lub+
Escrutinio Anticuerpos Irregulares	NEGATIVO
COOMBS DIRECTO	NEGATIVO

A.B.F. Firma

27/02/2025 13:19 CENTRO DE TRANSFUSION DE TOLEDO (A.B.F.) 1 / 1

Figure S5. Original report on red blood cell phenotyping in a whole blood sample from a man donor included in the BioGBS study. Donor data are hidden for anonymization reasons.
